# The duality of microchimerism and cancer in parous women: a review and evolutionary perspective

**DOI:** 10.1007/s00281-025-01041-0

**Published:** 2025-02-13

**Authors:** Cristiano Parmeggiani, Katja Sallinger, H. James Cleaves, Amy M. Boddy

**Affiliations:** 1 Department of Anthropology, University of California, Santa Barbara, CA 93106-3210 USA; 2 Division of Cell Biology, Histology and Embryology, Medical University of Graz, 8010 Graz, Austria; 3 Department of Chemistry, Howard University, Washington, DC 20059 USA; 4 Blue Marble Space Institute of Science, 600 1St Avenue, 1St Floor, Seattle, WA 98104 USA

**Keywords:** Microchimerism, Cancer, Pregnancy, Genomic conflict, Immune surveillance

## Abstract

The transfer of a small number of cells between parent and offspring during pregnancy, commonly referred to as microchimerism, is thought to occur in all human pregnancies. The impact of microchimeric cells on health outcomes in mothers and offspring with respect to cancer, remains unknown. Molecular and epidemiological studies yield conflicting results on the link between microchimerism and cancer, underscoring the complexity of this phenomenon. Further, most studies on microchimerism and cancer focus on the relationship between circulating fetal cells in parous women. Given that the cellular exchange between the mother and offspring is thought to have arisen due to the evolution of internal gestation, we provide an evolutionary perspective on how internal gestation may impact the risk of cancer in humans. We highlight the potential mechanisms that may play a role in cancer vulnerability in mammals, such as genomic conflict and placental invasion. We then review the literature to investigate the effects of microchimerism on cancer outcomes in parous women, highlighting each study's interpretation of the role microchimeric cells play in cancer development, whether it is a protective or contributing role. We conclude that our current understanding of the relationship between microchimerism and cancer is poorly understood and propose mechanisms for when we would expect to see microchimerism contribute to a role in protecting the host from cancer and when microchimerism may contribute to tumor development. Future studies, including more advanced methods to detect and identify microchimerism, will be important for elucidating the link between microchimerism and cancer initiation and progression.

## Introduction

Cancer is one of the main causes of death among humans [1]. There are many factors that influence or mediate cancer risk, including age, sex, and environmental conditions. For instance, men have an incidence rate for non-sex-specific cancers that is two to three times higher than that of women [2, 3]. It is generally believed that the phenomenon of cancer is the largely inevitable result of multicellularity: given enough cells, cell divisions, and somatic mutations, mutant cells occasionally arise. These mutant cells evade normal cell-cycle regulation checkpoints and the immune system to give rise to neoplasms, which are defined as abnormal growths of cells [4, 5]. Neoplastic cells can stay benign, with distinct borders, and benign tumors are typically not life threatening. However, some neoplastic cells gain the ability to invade the surrounding tissue, which defines malignant cancer. Cancer cells also can metastasize and implant in novel locations, with their spread often, but not exclusively, facilitated by the circulatory system. Metastatic cancer is a life-threatening disease, and it is estimated that the majority of patients that die from cancer die from metastatic disease.

During mammalian pregnancy, there is a bidirectional transfer of cells between the mother and fetus, which can result in the phenomenon of microchimerism [6]. We define cellular microchimerism as the presence of a relatively small number of cells from a genetically distinct individual in the body of another genetically distinct individual *of the same species*. Microchimerism invasion into the host may have some parallels with cancer metastasis. Microchimeric cells persist for decades after pregnancy and proliferate in the host body. While microchimeric cells may exhibit some phenotypes of cancer, such as migration and evading the host immune system, there is currently no evidence to suggest they are cancer-like in behavior or function. However, there is a debate in the literature about whether microchimeric cells can contribute to diseases, such as cancer, or if they play a protective role.

In this review, we explore the link between cancer and microchimerism, and propose new arguments for when one might expect to see a link between the two. We first discuss cancer vulnerabilities across animals from an evolutionary perspective, as comparative data suggest mammals are more susceptible to cancer than other taxa, suggesting a possible link between internal gestation and cancer risk. We then review the literature on cancer and microchimerism. We report mainly on male-origin cells' contribution to cancer in parous women due to limitations with studying microchimerism and cancer in other host systems. Lastly, we explore potential theoretical connections between cancer, pregnancy, and microchimerism, proposing a dual role for microchimerism in cancer, including contributing to the tumor microenvironment and/or protecting the host via immune surveillance. These insights may help explain how mammals manage the challenges of integrating adaptive immune systems with the multigenerational legacy that arises from internal gestation.

## Cancer from an evolutionary perspective

### Multicellularity and mammals

Cancer is a common disease among multicellular organisms, occurring across taxa from invertebrates [7] to vertebrates [8], and even in dinosaur fossils [9]. Comparative approaches to estimate cancer prevalence show that mammals have higher rates of neoplasia and malignant cancers compared to other vertebrates. Effron et al. [10] were among the first to report this pattern. They found the presence of neoplasia in animal necropsies in 2.75% of mammals, 1.89% of birds, and 2.19% of reptiles. Compton et al*.* [8] reported results from a wider dataset, with a mean prevalence of neoplasia and cancer at death that was highest in mammals (neoplasia = 15.28%, malignancy = 9.82%;), followed by birds and reptiles (neoplasia = 6.40%, malignancy = 4.33%) and amphibians (neoplasia = 4.16%). However, another study on cancer across birds, reptiles, and mammals report mammals are a close second (7% neoplasia, 4% malignancy) to squamates (*i.e.* reptiles) in malignancy prevalence [11]. The overarching presence of cancer across vertebrates, and the variation in cancer rates across taxa suggests that cancer is an evolutionary force affecting natural selection [12]. Notably, gestation time is negatively correlated with neoplasia and malignant prevalence, [8] and cancer mortality [13], suggesting that longer gestation periods reduce cancer risk.

### The placenta is a conduit for cellular exchange

Mammals produce a transient organ, the placenta, that is essential for communication between the maternal body and the developing fetus. Placental morphologies range from different depths of invasion and maternal–fetal tissue integration [14]. Placentation also represents a distinctive evolutionary adaptation that can facilitate the exchange of microchimeric cells. Micromicherism is common in placental mammals [15], having been observed in many species with different placental morphologies, including primates [16], mice [17, 18], dogs [19], and cows [20]. During pregnancy in placental mammals, the cells of two genetically close but distinct organisms, each sharing ~ 50% of their DNA, reside in proximity for significant periods of time with direct blood contact [21]. Researchers have noted for some time that trophoblasts, the cells forming the outer layer of the placenta, exhibit many characteristics commonly associated with cancer cells, including the ability to promote inflammation and angiogenesis, the evasion of immune destruction and apoptosis, and the promotion of cell mobility and invasion [21–23]. This pattern is observed consistently across different types of cancer, suggesting a fundamental biological connection between the function of the placenta and the mechanisms of cancer. However, the causal mechanisms behind such a high cancer rate among mammals and the role played by placental invasiveness in cancer onset remain open questions, with varied findings in the literature [13, 24–26].

### The placenta can lead to genomic conflict (and cancer)

From an evolutionary theory perspective, the interests of the fetus and the mother diverge due to their distinct genetic compositions, as each individual is driven by different evolutionary pressures. While the mother and fetus share common goals related to survival and reproduction, the fetus may demand more resources than the mother can provide. This is known as maternal–fetal conflict or genomic conflict [27]. The theory of genomic conflict suggests genetic actions beneficial for the transmission of fetal genes, such as strategies to extract more resources from the mother than what is optimal for health, can lead to competition between the mother and fetus. This competition might adversely affect the health of the mother or fetus, as advantageous traits for one can be detrimental to the other.

Genomic conflict may also lead to cancer vulnerabilities in mammals. For example, trophoblast cells evolved to extract resources from maternal tissue. Similar to cancer-like cells, trophoblast cells evade the maternal immune system and can invade local tissues. The evolutionary adaptations that facilitate trophoblast cells to transfer resources efficiently to the fetus can also increase the risk of cancer. Further, these evolutionary dynamics can lead to intragenomic conflict between the maternal and paternal genes, also leading to cancer vulnerability [28]. This complex interplay between cooperation and conflict during pregnancy reflects the broader evolutionary pressures that shape the health and survival of both mother and fetus and provides a lens into the ultimate role microchimerism may play in host biology. In other words, microchimerism is likely to contribute both positive and negative impacts on the host, depending on the context of the environment, including factors such as immune system dynamics, specific tissue where the microchimeric cells are located, if there are multiple generations of microchimeric cells that interact, and age and health of the host.

### Evolutionary vulnerabilities for cancer risk and pregnancy

The deep connection between the mother and developing fetus is an evolutionary trait that provides many advantages, but can also present vulnerabilities. For example, the transmission of maternal cancers to fetuses is known to occur. The converse (transmission of fetal cancers to mothers) is rare, since cancer incidence itself is largely age-related. Maternal cancer during pregnancy is diagnosed in between 0.02% and 0.1% of all pregnancies. The predominant cancers during pregnancy include breast and cervical cancers, malignant melanomas, and lymphomas [29], and once transmitted to the fetus, it is diagnosed as the same cancer type afflicting the mother [30]. When metastatic disease occurs in mothers during pregnancy and is transmitted to the fetus, the placenta is the most commonly affected tissue, with about 84.7% of reported cases [31], suggesting the placenta is a transient barrier that limits some cellular exchange.

Choriocarcinoma is another notable example of cancerous chimeric cell transfer, in this case from the fetus to the mother. Choriocarcinoma is an aggressive cancer derived from placental fetal trophoblasts, and it is the most common among true gestational trophoblastic neoplasms [32]. This cancer is derived from epithelial tissue, and tends to develop early systemic metastasis [33]. Conversely, hydatidiform moles, which are initially benign, are often the product of a fertilized egg containing only paternal chromosomes [34]. These non-viable eggs implant in the uterus and develop into tissue masses. In extreme cases, these masses can transform into invasive choriocarcinoma in the mother, sometimes metastasizing in both mother and fetus [34–36]. Choriocarcinomas appear to evade maternal immune rejection by hijacking a feature of trophoblastic cells, *i.e.*, by not expressing human leukocyte antigen (HLA) Class I or Class II antigens [36–38].

Beyond pregnancy, reproductive history, including the number of pregnancies and the age at which these occur, significantly impacts the risk of various types of cancers in parous women. This suggests that parity and pregnancy-related factors involve a complex interplay of hormonal and biological changes that can either increase or decrease cancer risk, depending on the specific context [39–45]. Epidemiological studies strongly support a link between reproduction and cancer, but the underlying mechanisms remain unclear, largely because factors like maternal health, fertility, and age—often confounding variables—are rarely fully considered.

## Microchimerism and cancer in humans

**Fig. 1:**
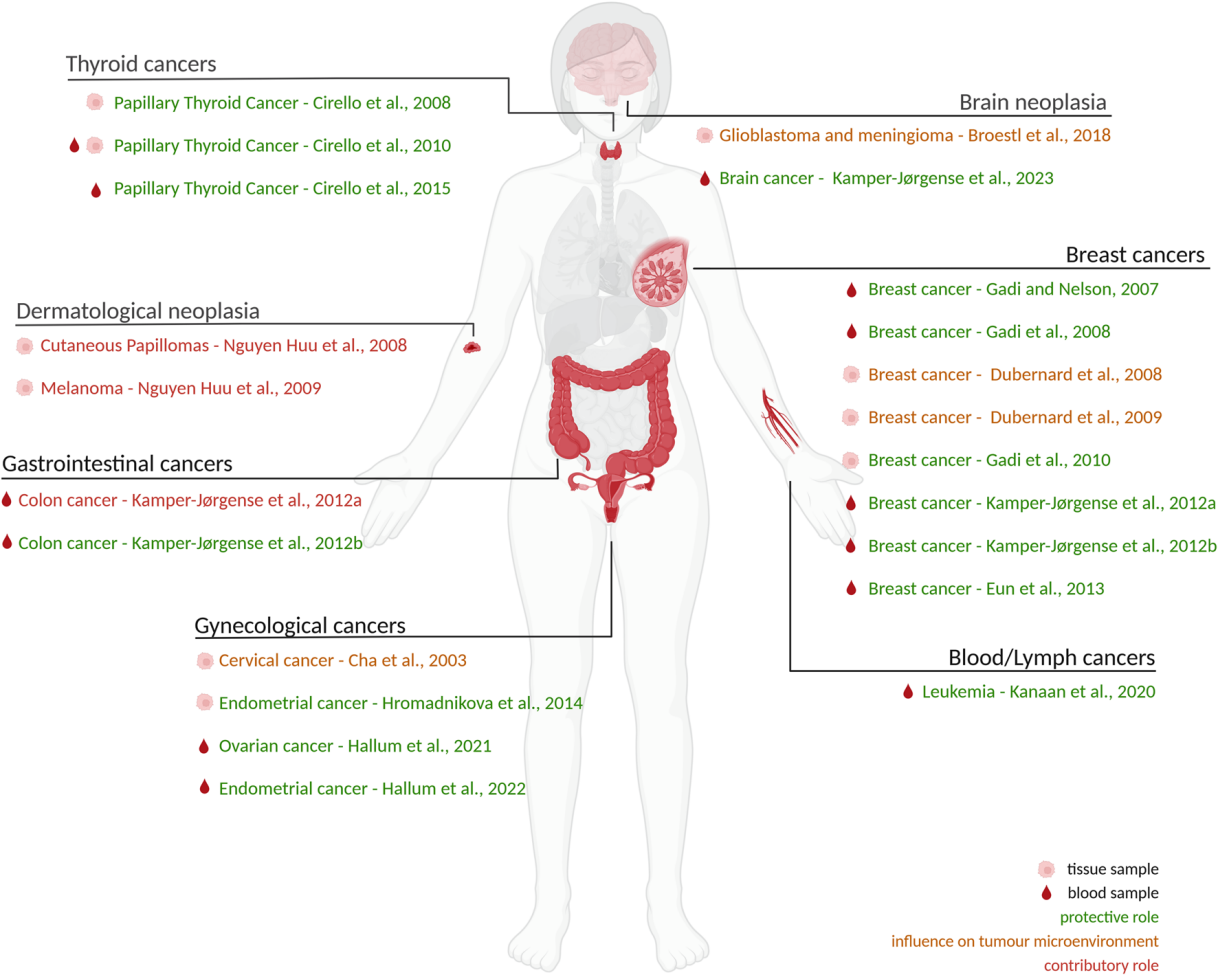
Summary of the current literature reporting an association between microchimeric cells and cancer, organized by cancer diagnosis. The organs include the brain, breasts, blood/lymphatic system, thyroid, skin, gastrointestinal tract, and reproductive organs. Each highlighted organ is annotated in Table 1. Additionally, we have highlighted the tissue type tested, including peripheral blood or the tumor tissue. Studies report either a protective role for microchimeric cells (green), contributory role (red), or an influence on the tumor microenvironment (orange). Figure generated with BioRender

The presence of microchimerism has been documented in a variety of human cancers in several studies (Fig. 1, Table 1), highlighting its potential influence on tumor biology. Microchimeric cells have been identified in several types of tumors, including breast [46–50], thyroid [51–53], glioblastoma and meningioma [54], ovarian [55], cervical [56] and skin [57, 58] cancers as well as in leukemia [59]. Recent reviews have highlighted how male-origin microchimeric cells can contribute to both tissue repair and tumor development, emphasizing the dual nature of their influence [60]. However, the mechanisms underlying these interactions remain incompletely understood.

**Table 1:** Detailed summary of studies which have associated microchimerism with cancer. Most studies have reported on fetal microchimerism in cancer; however, since detection commonly relies on probing for the Y chromosome, we have opted to label it as "male microchimerism." The column, "Potential role in cancer", reflects the roles suggested by the authors of each study. MC = microchimerism; polymerase chain reaction (PCR); fluorescence in situ hybridization (FISH); immunohistochemistry (IHC)

Author and year	Cancer Type	Type of MC, species and sample type	Number of individuals	Methods to Detect MC	Quantification	Results	Potential role in cancer	Statistics
Cha et al*.* (2003)	Cervical Cancer	Male MC, human, tissue	15 women (8 with cervical cancer and 7 controls)	FISH for X and Y chromosomes and IHC	MC cells detected in all patients with large section samples, less in smaller biopsies, none in control group	Male MC detected in all large samples. Fetal cells were either positive for CD45 or cytokeratin, but none of them for both markers	influence on tumor microenvironment	No statistical analysis was performed
Gadi and Nelson et al*.* (2007)	Breast Cancer	Male MC, human, blood	82 women (35 with breast cancer, 47 healthy controls)	Quantitative PCR for Y-chromosome-specific gene DYS14	MC cells found in 43% of healthy women vs. 14% of women with breast cancer	Lower prevalence MC in breast cancer patients suggests a protective role. It may provide allogeneic immune surveillance against malignant cells	protective	Odds ratio 4.4 (95% CI, 1.34–16.99; p = 0.006)
Dubernard et al*.* (2008)	Breast Cancer	Male MC, human, tissue	14 women	FISH for X and Y chromosomes, and IHC	MC cells were detected in 90% of breast cancer samples, with an average frequency of 36 fetal cells per 10^6^ maternal cells	Male MC cells in tumor stroma, expressing mesenchymal markers, suggest a role in cancer behavior and raise the potential of targeting them to affect tumor progression	influence on tumor microenvironment	Mean MC count: 19 fetal cells/10^6 maternal cells; p < 0.01
Gadi et al*.* (2008)	Breast Cancer	Male MC, human, l blood	99 women (54 with breast cancer and 45 healthy controls)	Quantitative PCR for Y-chromosome-specific gene DYS14	MC more common in healthy women (56%) than in those with breast cancer (26%)	Lower prevalence of MC in the blood of breast cancer patients suggests a protective role	protective	Odds ratio (OR) 0.29 (95% CI, 0.11–0.83; p = 0.02)
Nguyen Huu et al*.* (2008)	Skin Tumors (Cutaneous Papillomas)	Male MC, mouse, tissue	16 (12 transgenic mice and 4 controls)	Breeding of EGFP-expressing transgenic mice and FISH for X and Y chromosomes	MC cells identified in 9 out of 12 tumors (75%), absent in normal skin	Indicates recruitment of MC cells in early skin carcinogenesis with various cell markers, indicating potential influence on tumor progression, contrary to protective role hypothesis	contributory	Tumors: 75% contained fetal cells vs. 0% in normal skin (p < 0.001). Mean fetal cells in tumors: 90 vs. 0 per million maternal cells (p < 0.01)
Cirello et al*.* (2008)	Papillary Thyroid Cancer (PTC)	Male MC, human, tissue	63 women (40 PTC with male pregnancy, 23 PTC with female pregnancy or nulliparous) and 10 male controls	PCR of the male-specific sex-determining region Y gene, FISH for X and Y chromosomes and IHC	MC present in 47.5% of PTC cases among women with male pregnancies, with microchimeric cell counts ranging from 1.8 to 7.8 in tumor tissues and 0 to 1.5 in normal tissues	MC may serve a protective function, with some microchimeric cells (CD45 + /MHCII-) potentially attacking tumor cells, while others (Tg + /MHCII-) may contribute to tissue repair	protective	The number of maternal cells was significantly higher in neoplastic thyroid tissue than normal sections (p = 0.02, by Mann–Whitney rank sum test)
Dubernard et al*.* (2009)	Breast Cancer	Male MC, mouse, tissue	9 breast carcinomas from pregnant mice, and liver control samples from same set	FISH for X and Y chromosomes	20 fetal cells per 10^6^ maternal cells in breast carcinomas versus 4.9 per 10^6^ maternal cells in liver tissues	MC cells were present in all carcinomas, with higher concentrations in high-grade tumors. Although they express cytokeratin, they potentially contribute to tumor stroma and affect tumor progression	influence on tumor microenvironment	Mean MC count: maternal cells in tumors vs. fetal cells in liver (p = 0.011); high-grade: 29 vs. low-grade: 13.8 fetal cells/10^6 (p = 0.032)
Nguyen Huu et al*.* (2009)	Melanoma	Male MC, human and mouse, tissue	23 mouse samples and 31 human samples (16 melanoma samples, 8 nevus samples and 7 skin biopsy of polymorphic eruptions)	Breeding of EGFP-expressing transgenic mice, FISH and IHC	MC cells in 63% of human melanomas, 12% in benign nevi, 57% in mouse B16 melanomas	MC cells contribute to angiogenesis and lymphangiogenesis in melanomas (50% of cells expressed CD34, CD31, or von Willebrand factor), potentially influencing tumor growth during pregnancy	contributory	Human: 63% melanomas with fetal cells vs. 12% nevi (p = 0.034). Mice: 56% melanomas with fetal cells vs. 0% in normal skin (p < 0.01)
Cirello et al*.* (2010)	Papillary Thyroid Cancer (PTC	Male MC, human, l blood and tumor tissues	57 patients with PTC and 49 controls and neoplastic tissue from 19 women	PCR for Y-chromosome-specific gene DYS14, FISH	MC detected in 28/57 patients in blood (49.1%) vs. 38/49 controls (77.6%); MC detected in 6/19 tumors (2.1–6.9 cells/section)	MC was significantly lower in blood of PTC patients compared to controls. MC detected in tumors but absent from blood in some patients	protective	Mean MC count: PTC patients: 49.1% vs. Controls: 77.6% (p = 0.002)
Gadi et al*.* (2010)	Breast Cancer	Male MC, human, tissue	38 women (19 with breast cancer and 19 healthy controls)	Quantitative PCR to detect the Y chromosome-specific gene DYS14	MC was identified in 63% of healthy women and in 26% of women with breast cancer	MC was detected with greater frequency in breast tissue from women without cancer than in normal tissue near invasive tumors in those with cancer	protective	Odds ratio 0.17 (95% CI, 0.04–0.76; p = 0.02)
Kamper- Jorgensen et al*.* (2012a)	Breast Cancer and Colon Cancer	Male MC, human, blood	428 patients (89 breast cancer samples, 67 colon cancer samples and 272 healthy controls)	Quantitative PCR to detect the Y chromosome-specific gene DYS14	MC detected in 70% cancer-free women, 40% of women who later developed breast cancer, and 90% of women who later developed colon cancer	Lower prevalence of MC in women who developed breast cancer suggests a potential protective role, while higher prevalence in colon cancer points to a contributory role in cancer development	protective for breast cancer and contributory for colon cancer	Breast cancer: Odds Ratio (OR) = 0.30 (95% CI: 0.17–0.52). Colon cancer: OR = 3.9 (95% CI: 1.6–9.5)
Kamper- Jorgensen et al*.* (2012b)	Breast Cancer and Colon Cancer	Male MC, human, blood	89 breast cancer cases, 67 colon cancer cases	Quantitative PCR to detect the Y chromosome-specific gene DYS14	Breast cancer: 95.8% 10-year survival in microchimerism-positive vs. 88.6% in negative women Colon cancer: No significant difference	Presence of MC was associated with better survival in breast cancer cases, but no clear effect in colon cancer survival	protective	Breast cancer Hazard Ratio (HR) = 0.34 (95% CI: 0.06–1.78). Colon cancer HR = 0.89 (95% CI: 0.20–3.92)
Eun et al*.* (2013)	In Situ Breast Cancer (CIS)	Male MC, human, blood	89 CIS patients, 88 healthy controls	Quantitative PCR for DYS14 gene	MC detected in 85% of controls vs. 64% of CIS patients	Deficiency of MC is associated with higher risk for CIS, suggesting a protective role via immune surveillance	protective	Odds ratio for protection against CIS = 0.26 (95% confidence interval 0.12–0.56; p = 0.001)
Hromadnikova et al*.* (2014)	Endometrial Cancer (EC)	Male MC, human, tissue	75 women with endometrial cancer (EC) (47 type 1 EC, 28 type 2 EC), 41 controls	Quantitative PCR to detect the Y chromosome-specific gene DYS14	MC detected in 38.3% of type 1 EC patients vs. 70% of controls	MC levels significantly lower in women with type 1 EC compared to benign conditions. Lower MC prevalence associated with better prognosis	protective	Odds Ratio (OR) = 0.257 (95% CI: 0.105–0.628, p = 0.003)
Cirello et al*.* (2015)	Papillary Thyroid Cancer (PTC)	Male MC, human, l blood	87 parous women with PTC, 66 healthy controls, 57 nonparous PTC	PCR of the male-specific sex-determining region Y gene	MC in 39.1% of PTC patients vs. 63.6% in healthy controls	MC + patients had lower extrathyroidal extension (p = 0.027), lower lymph node metastases (p = 0.044), and higher remission rates (94.1% vs. 67.9%, p = 0.009)	protective	MC detection: Healthy controls: 63.6% vs. PTC: 39.1% (p = 0.004)
Broestl et al*.* (2018)	Brain Tumors (Glioblastoma and Meningioma)	Male MC, human, tissue	60 patients (32 glioblastoma, 25 meningioma and 3 control samples)	Quantitative PCR for male DNA and FISH for X and Y chromosomes	MC occurred in 78.1% of glioblastomas and 48% of meningiomas, with cell frequencies ranging from less than one to 46 per 10^5 cells	MC is present in about 78.1% of glioblastoma and 48% of meningioma cases, but it shows no clear correlation with the standard clinical or molecular markers of these conditions	influence on tumor microenvironment	MC prevalence significantly higher in GBM than meningioma (p < 0.05, Fisher's exact test)
Kanaan et al*.*(2021)	Leukemia	Maternal MC, human, blood	HLA-genotyping of 68 cord blood (CB) mothers, with 36 patients evaluated across multiple time points after exclusions	HLA-specific quantitative PCR targeting polymorphisms unique to CB mother	CB-maternal MC was present in 7 patients (~ 20%)	CB-maternal MC is linked to a lower risk of leukemia relapse after cord blood transplantation (CBT), detectable up to a year post-transplant. Leukemia relapse rate after CBT is reduced more than twofold compared to HLA-matched or mismatched donors, suggesting CB-maternal MC might improve immune tolerance and post-transplant outcomes	protective	Probability of relapse for cord blood positive vs. cord blood negative individuals: (p = 0.1691)
Hallum et al*.* (2021)	Ovarian Cancer	Male MC, human, blood	700 women (100 with ovarian cancer, 600 control)	Quantitative PCR to detect the Y chromosome-specific gene DYS14	Male microchimerism detected in 46% of cases vs. 65.9% of controls	An association between MC and a decreased risk of ovarian cancer suggests a protective effect, with potential roles in tissue repair and wound healing	protective	MC positive women: reduced hazard ratio (HR) of ovarian cancer compared with women testing negative (HR = 0.44, 95% CI: 0.29–0.68)
Hallum et al*.* (2022)	Type 1 Endometrial Cancer (EC)	Male MC, human, blood	76 type 1 EC cases, 505 controls	Quantitative PCR for Y-chromosome (DYS14)	MC detected in 65.9% of controls vs. 54.0% of type 1 EC cases	Reduced rate of Type 1 EC, but not other types of endometrial cancers	protective	Reduced rate of Type 1 endometrial cancer (HR = 0.66, 95% CI: 0.39–1.00)
Kamper- Jorgensen et al*.* (2023)	Brain Cancer	Male MC, human, blood	73 cases, 505 controls	Quantitative PCR for Y-chromosome (DYS14)	MC detected in 46.5% of cases and 65.9% of controls	MC-positive women had a 50% reduced risk of brain cancer	protective	Hazard Ratio (HR) for BC: 0.50 (95% CI: 0.33–0.77); high-grade gliomas HR: 0.47 (95% CI: 0.29–0.75); low-grade gliomas HR: 0.52 (95% CI: 0.15–1.79)

To further explore the relationship between microchimeric cells and cancer, we conducted a literature review utilizing the Microchimerism Literature Atlas (https://literature-atlas.microchimerism.info/). For our review, we set the filter to "Cancer" within the Literature Atlas, yielding a list of 575 entries. We then selected studies with titles containing both "microchimerism" and "cancer." Subsequently, we excluded review papers, studies involving non-human samples (e.g., dog models), and one paper that did not focus on a specific cancer type. Additionally, we included five relevant studies (Dubernard, Nguyen Huu, and Kanaan) that were not captured by the Literature Atlas search. This resource allowed us to identify and analyze studies investigating microchimerism in various cancer types. Microchimerism studies employ various detection techniques, including polymerase chain reaction (PCR) for identifying male-specific DNA sequences, fluorescence in situ hybridization (FISH) for X and Y chromosomes, and immunohistochemistry (IHC) staining. Because of the technical methods used to detect microchimerism, most studies have concentrated on identifying fetal cells in the blood or tissues of women who have given birth. Here, we note that most studies aiming to identify fetal cells rely on Y chromosome markers, making it impossible to differentiate between fetal cells and those originating from male microchimerism. To maintain technical accuracy, we will report male microchimerism rather than fetal microchimerism when the detection method relies strictly on Y chromosome.

### Gynecological cancers

In gynecological oncology, the presence of male-origin microchimerism has been investigated in several studies, which suggest a complex and sometimes contradictory role for microchimerism in cancer of parous women. In a study conducted by Cha et al*.* [56], male-origin microchimeric cells were identified within cervical cancer tissues of all patient samples when analyzed using large section samples (1.5 × 2 cm), and in smaller quantities in those with smaller biopsy specimens (0.1 × 0.5 cm). These male-origin cells were not detected in any tissue specimens from the control group. Within cervical cancer tissues, it was observed that identified male-origin cells express markers such as CD45 or cytokeratin. CD45 is a marker of hematopoietic (immune) cells, indicating that some male microchimeric cells may be of immune origin and potentially involved in immune responses within the tumor. Cytokeratin is an epithelial cell marker, suggesting that other male microchimeric cells may differentiate into epithelial-like cells within the tumor tissue. However, the impact of these cells on the progression of the disease has yet to be fully elucidated. Hallum et al*.* [55] found an inverse relationship between microchimerism presence and ovarian cancer risk by analyzing blood samples from 700 women for Y chromosome presence, indicative of male microchimerism. The study revealed that male microchimerism was detectable in 46% of ovarian cancer cases and 65.9% of controls. Furthermore, women who tested positive for male-origin microchimerism had a significantly reduced risk of developing ovarian cancer, evidenced by a hazard ratio of 0.44, indicating a protective effect.

In addition to studies on cervical and ovarian cancers, research by Hromadnikova et al*.* [61] investigated the presence of male microchimerism in endometrial tissues. Their study demonstrated that microchimeric cells are commonly found in benign uterine disorders but are less prevalent in uterine cancers, suggesting a protective role of microchimeric cells in benign tissue. However, of those individuals with uterine cancer, a lower prevalence of male-origin microchimerism was associated with better prognosis. Further expanding on the role of microchimerism in endometrial cancer, Hallum et al*.* [62] reported that microchimerism was present in the peripheral blood of 65.9% of the controls but only in 54.0% of women with endometrial cancer, lending additional support that circulating levels of male-origin microchimerism may play a protective role. Both Hromadnikova et al*.* [61] and Hallum et al*.* [62] found no relationship between the concentration of male-origin microchimerism and cancer risk. Overall, the results of these studies suggest the presence of microchimerism in the blood or benign tissues may have a protective effect for endometrial tissues, however the histological grade and stage of the tumor should be considered for future studies.

### Breast cancer

Several studies have investigated the link between male-origin microchimerism and breast cancer, suggesting a potential protective role against the development of breast cancer. These studies have utilized different methodologies and sample types, including peripheral blood and breast tissue, across human and murine models.

Gadi et al*.* conducted case–control studies analyzing the presence of male-origin microchimerism in peripheral blood from women with and without breast cancer. Using real-time quantitative PCR targeting the Y chromosome-specific gene *DYS14*, they consistently found that male-origin microchimerism was more prevalent in healthy women compared to those with breast cancer [48, 63]. For instance, one study revealed microchimeric cells in 56% of healthy controls but only in 26% of women who developed breast cancer, yielding an odds ratio of 0.29, indicating a significantly lower risk of developing breast cancer among women with detectable microchimerism [48]. Another analysis found similar trends, with 43% of healthy women testing positive for male-origin microchimerism compared to only 14% of breast cancer patients [63]. These findings suggest that male-origin microchimerism may serve as a protective mechanism, possibly through enhanced immune surveillance against malignant cells [48, 63]. Building upon these findings, Gadi [49] applied quantitative PCR targeting the Y chromosome gene *DYS14* to analyze breast tissue from women with and without breast cancer. This study revealed a higher prevalence of microchimeric cells in the breast tissue of women without cancer compared to that near invasive tumors in breast cancer patients. Specifically, male-origin microchimerism was found in 63% of healthy breast tissues but only in 26% of tissues adjacent to invasive disease in women with breast cancer. This result provides further support to the notion of male-origin microchimerism's protective properties against breast cancer, suggesting that the protective association extends beyond the peripheral blood into the breast tissue itself. Adding to these perspectives, Eun et al*.* extended these findings to in situ breast cancer (CIS), analyzing blood samples from 89 CIS patients and 88 healthy controls using quantitative PCR for the DYS14 gene. They detected male-origin microchimerism in 85% of healthy controls but only in 64% of CIS patients, indicating a deficiency of microchimerism in patients with CIS [64]. Kamper-Jørgensen et al*.* [50] conducted a study examining the presence of male microchimerism in women with and without breast cancer. They observed that male microchimerism was more prevalent in women without breast cancer than those with the disease, suggesting microchimerism could have a protective role. Specifically, they found male microchimerism in 70% of cancer-free women compared to 40% of women who later developed breast cancer. This finding corroborates the earlier results by Gadi et al., reinforcing the hypothesis that male microchimerism could serve as a protective mechanism against the development of breast cancer.

In contrast, Dubernard et al. [46] analyzed breast tissue samples from women with breast cancer that developed during pregnancy and compared them to benign mammary lesions. They found that male-origin cells were present in 90% of breast carcinomas but were absent in all benign lesions examined. The microchimeric cells were primarily located in the tumor stroma, which plays a critical role in tumor proliferation and progression. The fact that these cancers developed during pregnancy may indicate that the influx of microchimeric cells during this period could contribute to tumor development or influence the tumor microenvironment, potentially promoting cancer progression. Expanding on their earlier work, Dubernard et al. [47] studied male-origin microchimerism in high-grade breast carcinomas occurring during pregnancy using a murine model. They observed a higher concentration of male-origin cells in high-grade tumors compared to control tissues. While the presence of microchimerism in tumors might suggest a contributory role to tumor progression, the authors acknowledged that these cells could have dual roles, proposing that male-origin microchimerism could contribute to the tumor stroma and potentially promote tumor growth.

These studies collectively indicate that microchimerism’s role in breast cancer is complex and may vary based on pregnancy status, tissue type, and the tumor environment. Research by Gadi et al. and Kamper-Jørgensen et al. suggests a protective effect of male-origin microchimerism when found circulating in the blood, particularly in non-pregnant women. In contrast, findings by Dubernard et al. show that male microchimerism within tumor tissue may contribute to cancer progression during pregnancy, suggesting these cells play different roles in the blood vs the tumor tissue. This emphasizes the need for further research to clarify the conditions under which male microchimerism is protective versus when it may contribute to disease progression in breast cancer.

### Dermatological neoplasia

In dermatological oncology, two sequential studies by Nguyen Huu [57, 58] have explored the involvement of male-origin microchimerism in the development and progression of skin tumors, including both benign and malignant lesions. These studies provide valuable insights into how microchimerism may contribute to tumorigenesis, particularly during pregnancy. The first study [57] focused on cutaneous papillomas, which are benign skin lesions not classified as cancerous. Using a transgenic mouse model expressing Enhanced Green Fluorescent Protein (EGFP) and fluorescence in situ hybridization (FISH) to detect Y chromosome-specific sequences, the researchers tracked fetal cells within the tumors. They found a significant presence of male-origin cells in 75% of the papilloma tumors examined. In contrast, such cells were absent in normal skin samples from the same mice, suggesting a specific association of male-originl microchimerism with tumor tissues. The microchimeric cells expressed various cell markers, indicating their potential influence on tumor progression, which is contrary to the hypothesis of a protective role of microchimerism. Expanding upon these findings, the second study by Nguyen Huu et al*.* [58] investigated melanomas, malignant skin cancers representing a serious health concern. This study used both human and murine models, with a particular focus on melanomas that developed during pregnancy. The occurrence of melanoma during pregnancy is particularly concerning due to the potential for more aggressive disease progression. Employing both FISH and immunohistochemistry (IHC) techniques, the researchers detected fetal cells in 63% of human melanomas and 57% of mouse B16 melanomas. Notably, in 50% of these cases, the microchimeric cells expressed endothelial markers such as CD34, CD31, or von Willebrand factor. This expression is indicative of their involvement in angiogenesis, the formation of new blood vessels within the tumor environment—a key process in tumor growth and metastasis.

The examination of benign nevi (non-cancerous moles) further reinforces the contributory role of male-origin microchimerism and fetal cells were found in only 12% of these lesions [59]. The presence of microchimeric cells was significantly lower in benign compared to malignant melanomas, strengthening the hypothesis that microchimerism may play a key role in the malignant transformation or progression of skin tumors. The involvement of male-origin microchimerism in melanoma angiogenesis suggests a possible mechanism through which microchimeric cells might actively drive tumor growth and metastasis during pregnancy.

### Thyroid cancers

Cirello et al*.* [51] investigated the role of male-origin microchimerism in Papillary Thyroid Cancer (PTC) and its potential impact on the progression of the disease. In this study, tumor tissue was analyzed from 63 women who had male offspring before their cancer diagnosis. PCR amplification targeting the sex-determining region Y (SRY) gene revealed male cells in 47.5% of these cases. Further analysis using FISH showed significantly elevated male microchimeric counts in cancerous tissue compared to surrounding healthy tissue. Combined FISH and IHC identified microchimeric cells that expressed thyroglobulin—a protein exclusively synthesized by thyroid cells, often used as a marker for thyroid cancer detection and monitoring due to its role in the production of thyroid hormones, in both tumor and normal tissues. Notably, microchimeric cells stained CD45 positive were detected only in tumor sections, indicating a potential immune response role. Moreover, microchimeric cells negative for either CD45 or thyroglobulin markers were detected in both tumor and normal tissues, suggesting these cells might possess "progenitor-like" properties capable of transdifferentiation into different cell types. Further studies by Cirello et al*.* expanded on the role of male-origin microchimerism in PTC. In addition to their initial findings in thyroid tissues [53], they analyzed peripheral blood samples and found that higher levels of male-origin microchimerism were associated with less aggressive tumor characteristics and better clinical outcomes [52, 53]. Specifically, patients with detectable microchimeric cells had smaller tumor sizes, lower rates of lymph node metastasis, and improved prognosis. These results suggest that male-origin microchimerism may exert a protective effect in PTC, possibly through immune-mediated mechanisms or by influencing tumor biology in a way that suppresses aggressiveness.

### Brain neoplasia

Brain tumors, particularly glioblastomas and meningiomas, were the focus of Broestl et al*.*’s [54] study. This work used quantitative PCR to detect male DNA in tumor tissue samples from women who had previous male pregnancies, aiming to quantify the presence of male-origin microchimerism within these tumors. The presence of male cells within the tumors was further validated through FISH targeting X and Y chromosomes. Male-origin microchimerism was identified in approximately 80% of glioblastoma cases and 50% of meningioma cases, highlighting a significant occurrence of microchimeric cells in these brain tumors, suggesting a contributory role of male-origin microchimerism in glioblastomas and meningiomas. In contrast, Kamper-Jørgensen et al. [65] conducted a blood-based study, analyzing peripheral blood samples from a cohort of 73 brain cancer cases and 505 controls. They found male-origin microchimerism in 46.5% of cases and 65.9% of controls, with microchimerism-positive women showing a 50% reduced risk of brain cancer.

### Blood/Lymph cancers

In the context of leukemia, Kanaan et al*.* [59] explored the relationship between maternal microchimerism in cord blood transplantation and leukemia relapse risk. Cord blood transplantation is a medical procedure that uses hematopoietic stem cells derived from umbilical cord blood to reestablish bone marrow function in patients whose marrow has been compromised by disease or chemotherapy. Cord blood transplantation is considered an alternative to traditional bone marrow transplants, particularly beneficial for patients who lack a compatible bone marrow donor, as cord blood does not require as close a match. Using a cohort of 95 patients who received either double or single cord blood transplants, HLA-specific quantitative PCR assays were employed to detect the presence of cord blood-derived maternal microchimerism following transplantation and 36 patients from this group were chosen for detailed analysis. Cord blood maternal microchimerism was found in 19.4% of these patients in bone marrow and peripheral blood, with the presence of maternal microchimerism observed up to one year post-transplantation. Patients with identifiable cord blood microchimerism had significantly reduced rates of relapse, mortality, and treatment failure post-transplantation, suggesting a protective effect of maternal microchimerism.

### Gastrointestinal cancers

Studies on the role of microchimerism in gastrointestinal cancers, particularly colon cancer, have yielded contrasting results. Kamper-Jørgensen et al*.* [50] conducted an epidemiological study to investigate the association between male microchimerism and colon cancer. Using peripheral blood samples from women, they found male-origin microchimerism was more prevalent in women who later developed colon cancer compared to healthy controls. Specifically, male-origin microchimeric cells were detected in 70% of women who developed colon cancer versus 46% in controls. This suggests a potential contributory role of microchimerism in the development of colon cancer.

Expanding on these findings, Kamper-Jørgensen [66] investigated the impact of microchimerism on survival after a cancer diagnosis. In this study, they examined whether the presence of male microchimerism in peripheral blood affected survival outcomes in women diagnosed with breast or colon cancer. The results indicated that women with detectable male-origin microchimerism had improved survival rates for both breast and colon cancers. For colon cancer patients, the presence of male-origin microchimerism was associated with a significantly reduced mortality risk compared to those where it was not detectable. This suggests that the timing, either pre or post diagnosis matters in interpretation and potential role microchimeric cells play in colon cancer prognosis and development.

## Discussion

Microchimeric cells are transmissible cell lines from different organisms of the same species. The function of microchimeric cells within the host tissue is still unknown. Across 20 studies examining the associations between microchimerism and cancer, the findings are conflicting on whether these cells contribute to or protect against cancer in the host [6, 7, 52, 67]. Recent studies [60], have highlighted the complex roles of microchimeric cells in cancer development and progression, discussing how male-origin microchimeric cells can contribute to both tissue repair and tumor development, emphasizing the dual nature of their influence. Microchimeric cells are somehow privileged by the host immune system and have the ability to survive, proliferate and differentiate into host tissue. These cells may depend on many of the same immunological systems hijacked by cancer, yet there is no clear evidence to demonstrate a causal link between microchimeric cells and cancer.

From a genomic conflict perspective, we do not anticipate a clear answer on whether microchimeric cells help protect or contribute to cancer risk in the host. Previous predictions based on genomic-conflict theory [21] suggest more conflict between maternal and fetal cells (and potentially more contributions to cancer progression) in tissues that are important for resource distribution, such as the breast, thyroid and the brain. The current epidemiological papers on microchimerism and breast cancer in parous women report the opposite of these predictions, and report a mostly protective role in thyroid and breast cancer. While it is difficult to assess the role of microchimeric cells in cancer based on the methodological limitations we outline below, based on this review of the literature it is clear that the conflict hypothesis in microchimerism research may need refinement.

The type of sample collected may influence reports of microchimerism's role in cancer, potentially reflecting underlying biological processes. In general, the studies that measure circulating microchimeric cells in peripheral blood tend to associate with a “protective role” in cancer, while studies that detect microchimerism in the diseased tissue, more often report a “negative or contributory role”. We speculate that when microchimeric cells are detected circulating in the blood, they may function in immune surveillance; however, when present within tumor tissue, these cells could be co-opted by the tumor, potentially aiding in disease progression. This complexity highlights the need for research that accounts for the nuanced and multifaceted nature of these rare cell and host immune interactions. Direct comparisons between current studies are challenging, as microchimerism likely plays protective, contributory, or even bystander roles depending on various factors. Future studies should consider the context, including the individual's age and parity, the tissue of origin for the cancer (e.g., blood versus tumor tissue), timing of diagnosis, and the extent of genomic overlap between host and microchimeric cells (such as HLA concordance).

### Limitations in detecting microchimerism for cancer research

Current research on microchimerism in cancer employs a range of methodologies that make direct comparisons between studies challenging and we caution against inferring any causal relationships between microchimerism and cancer risk. Studies differ in sample sizes and cohorts, which can influence the statistical power and generalizability of their findings. Additionally, variations in study design and sample selection—for example, detecting microchimerism in peripheral blood vs. cancer tissue can lead to differing interpretations of the role of microchimeric cells in cancer biology. The techniques employed, such as PCR, FISH or IHC, also affect the depth of functional analysis possible.

For instance, some studies [48, 49] examined breast cancer by detecting microchimerism in peripheral blood using PCR to identify the presence of Y chromosome-specific genes. These studies suggest a protective role for male-origin microchimerism based on prevalence data. Similarly, Hallum et al*.* [55] used PCR on blood samples to suggest an inverse relationship between the presence of male-origin microchimerism and the risk of developing ovarian cancer, implying a protective effect. However, these studies primarily assessed the presence of microchimerism without exploring the functional roles of the microchimeric cells. Without functional analyses—such as examining how male-origin microchimeric cells interact with tumor cells or contribute to immune responses—the protective effects of microchimerism in breast cancer may be overestimated. The presence of microchimerism does not confirm that these cells actively contribute to cancer suppression. Future studies that include functional profiles, along with detection of microchimeric cells in both the circulating blood and tumor tissue, may help identify a causal role.

In this review, we report some studies have explored characterizing microchimeric cells using techniques like FISH and IHC to identify various markers on these cells to infer their functional role. Characterization of microchimeric cells in the tissue of women with cancer resulted in contradictory outcomes in cancer development. Results from two studies suggest that these cells may promote cancer progression in the host by affecting the tumor microenvironment, such as contributing to angiogenesis in melanoma [59, 61]. In contrast, Cirello et al*.* [53] identified microchimeric cells that expressed markers like thyroglobulin, suggesting these cells might play a role in immune response and tissue repair. The variation in methodology and sample sources (e.g., systemic samples like blood versus localized tumor tissue) highlights an important point: distinct biological processes may be involved, and comparisons across studies using different sample sources should be avoided. In other words, a study measuring microchimerism in the blood of a cancer patient should not be directly compared to one that focuses on microchimerism localized in the tumor or the tumor microenvironment.

Further, studies that report on the presence or absence of microchimerism, or the quantity of microchimerism, should use caution when comparing to studies that attempt to characterize the microchimeric cells in the host tissue. Microchimeric cells may play different roles in various tissues, suggesting that their impact on cancer may vary depending on the tissue of origin. This means that microchimerism's role in cancer could differ across different types of cancer, making direct comparisons between them inappropriate. For example, Gadi et al*.* [48] found more male-originmicrochimeric cells in healthy tissues compared to breast cancer tissue, yet Nguyen Huu et al*.* [58] found male-origin microchimeric cells associated with angiogenic processes in melanomas. Microchimeric cells' role in cancer may depend on the unique conditions of the tissue of origin and tumor microenvironment, complicating any simple classification of microchimerism as solely protective or contributory.

While most studies infer identification of fetal cells through various methods discussed above, there are limitations in the ability to confidently ensure these cells are from fetal origin. The studies by Yan et al*.* [68] and Müller et al*.* [69] suggest that the detection of Y-chromosome DNA using PCR does not definitively indicate fetal microchimerism from a male child. Yan et al*.* [68] found male-origin microchimerism in 21% of women without sons, suggesting alternative sources such as unrecognized spontaneous abortions or vanished male twins. Similarly, Müller et al*.* [69] found male microchimerism in a cohort of healthy, nulliparous Danish girls, indicating possible trans-generational microchimerism where genetic material from a male relative could be transferred through generations via the maternal lineage or other familial pathways. Given this complexity, using PCR to detect Y-chromosome DNA as a standalone method may be insufficient for confirming fetal microchimerism. Employing more sophisticated genotyping techniques would allow researchers to more accurately determine the origin of microchimeric cells, such as genotyping or typing the HLA locus. Finally, it is important to recognize that quantifying DNA through PCR methods, although highly sensitive, does not guarantee that we are measuring intact cells; it may also detect fragmented DNA. This distinction is crucial, as the presence of fragmented DNA could lead to misinterpretations of the results regarding the viability and functional role of the microchimeric cells.

### The missing role of maternal microchimerism in cancer

Our current understanding of microchimerism in cancer is largely based on studies of parous women, due to methodological limitations in detecting microchimerism from non-fetal sources. The role of maternal microchimerism in cancer is a relatively underexplored area, and we report only one study by Kanaan et al*.* [59] in leukemia. Studying maternal cells in cancer biology can provide new research opportunities to examine cancer risk in both males and females. Maternal cells transferred to the fetus during pregnancy can persist into adulthood and are believed to exhibit various immunomodulatory and tissue repair functions that could also influence oncogenesis and cancer progression. Alternatively, these maternal cells may contain a repertoire of functioning immune cells that could protect the offspring from neoplastic progression. Future studies examining maternal microchimerism in the blood of both men (who have higher cancer incidence rates) and women, including those who are nulliparous, could help to address these identified knowledge gaps.

### Male-origin microchimerism may play a role in immune surveillance

Most studies reviewed here that identified male-origin microchimerism in the host’s blood reported a protective effect on the host’s physiology. Microchimeric cells, harboring both immunologic and stem cell-like properties, have the potential to enhance the immune response, potentially allowing the host to better identify and combat neoplastic cells. Immune surveillance is the biological process whereby the immune system mounts a defense against pre-cancerous and malignant cells, potentially leading to the elimination of transformed or malignant cells from the body [70, 71]. Despite the effectiveness of this natural defense mechanism, cancer manifests in individuals with seemingly intact immune systems. This phenomenon may be attributed to the occasional failure of the host immune system to completely eradicate newly transformed tumor cells during the initial stages of tumor development [70]. Microchimeric cells, which are known to possess both immunological and stem cell-like properties, are hypothesized to enhance immune surveillance by increasing the diversity of the maternal immune system [55, 72]. This diversity may enable a more effective immune response against cancer by potentially recognizing tumor antigens that could be overlooked by the host's regular immune cells. By contributing to a broader immune repertoire, microchimeric cells might aid in the detection and eradication of tumor cells. [55, 68]. Beyond immune surveillance, microchimeric cells can contribute to tissue repair and regeneration [73] thereby indirectly supporting the immune system's function in maintaining cellular integrity and responding to injury or disease [60].

This ‘immune surveillance’ theory is supported by observations in 10 examined cancer studies. For example, studies by Gadi et al*.* [48, 49] in breast cancer and Kanaan et al*.* [59] in leukemia, typically show a higher prevalence of microchimerism in healthy controls than in cancer patients, suggesting its potential protective role in systemic immune surveillance. An alternative explanation is that microchimeric cells may migrate from the bloodstream to tumor sites, reducing their detectability in the blood while increasing their presence in tissues. This migration could account for the contradictory results between blood and tissue analyses. Lastly, it is possible that these microchimeric cells are not directly involved in immune surveillance, but their detectability correlates with a heightened immune response in healthy individuals.

### Fetal microchimerism in tumor tissues may contribute to tumor microenvironments

Unlike cancer cells, male-origin microchimeric cells are not expected to have characteristics such as genomic instability, but they can still contribute to tumor biology. In contrast to the ‘immune surveillance’ theory of microchimerism and cancer, microchimerism may contribute to cancer microenvironment development, similarly to cancer associated fibroblasts (CAFs). More than half of the studies reviewed that identified microchimeric cells within tumor tissue suggested a possible role in disease progression.

CAFs are a key component of the tumor microenvironment, influencing cancer progression through support of tumor cell growth, remodeling of the extracellular matrix, and promoting angiogenesis and immunosuppression [74]. These cells facilitate an immunosuppressive environment that aids in the tumor's evasion of the immune response. CAFs secrete various cytokines and chemokines, which can recruit immune cells and suppress the natural immune response against tumor cells [75]. Studies suggest that male-origin microchimeric cells in cancerous tissues express markers characteristic of CAFs and might be involved in promoting angiogenesis and tumor progression. For instance, Dubernard et al. [47] found that these microchimeric cells localized within the tumor stroma of breast cancer tissues and expressed mesenchymal markers, suggesting their potential differentiation into CAFs and contribution to extracellular matrix remodeling. Similarly, Nguyen Huu et al. [59] reported that in melanoma tissues, male-origin microchimeric cells expressed angiogenic markers such as CD34, CD31, and von Willebrand factor, which could contribute to angiogenesis and lymphangiogenesis within the tumor microenvironment.

## Conclusion and Future Directions

Exploring the complex interplay between placentation, microchimerism, and cancer unveils a deeply interconnected relationship. This deep connection between the mother and baby may significantly impact oncological outcomes in eutherian (placental) mammals. The evolutionary development of placentation plays a critical role in the transfer of microchimeric cells into the mother and baby. This dyadic cellular exchange plays a positive role in reproductive success of both individuals, however, it may also lead to vulnerabilities among mammals, due to the interplay of genomic conflict. This direct biological connection between two individuals can facilitate the transfer of neoplastic and semi-allogeneic cells that may contribute to disease pathology. To better understand and explore the role of microchimerism in cancer initiation and progression, we propose two, non-mutually exclusive hypotheses, which will be dependent on the context of the analysis. Microchimerism may enhance immune surveillance, expanding the immunogenicity and providing a protective role in cancer biology of the host, especially when detected circulating in the blood of the host. However, microchimeric cells may also “follow the rules” of the body and contribute to neoplastic progression by being co-opted by the tumor microenvironment, as seen when male-origin cells were detected in tumor tissue Lastly, we highlight how the methodology of microchimerism detection in cancer biology is limited to which tissue and method is being employed. Future research on the functional role of microchimerism, along with comparative studies, may begin to elucidate the larger role these cells may play in cancer development and protection.

## Data Availability

Data sharing not applicable to this article as no datasets were generated or analysed during the current study.
